# Disability inclusion in malaria services in western Tanzania: A rapid barrier analysis

**DOI:** 10.4102/ajod.v12i0.1270

**Published:** 2023-11-28

**Authors:** Elias C. Nyanza, Anthony Kapesa, Theresia Maduka, Monica T. Madullu

**Affiliations:** 1 Department of Environmental, Occupational and Research GIS, School of Public Health, Catholic University of Health and Allied Sciences, Mwanza, Tanzania; 2 Department of Community Medicine, School of Public Health, Catholic University of Health and Allied Sciences, Mwanza, Tanzania

**Keywords:** malaria services, persons with disability, barriers, inclusion, vulnerable population

## Abstract

**Background:**

Persons with disabilities generally face greater challenges in accessing healthcare and interventions compared with the general population. Malaria is one of the diseases that can seriously affect individuals with disabilities, as it requires early diagnosis and prompt treatment.

**Objective:**

This study explores the extent to which locally available malaria services and interventions are inclusive of persons with disabilities and identifies associated access barriers.

**Method:**

A qualitative case study focusing on social, cultural and health system factors associated with the inclusion of persons with disabilities in malaria services was conducted in Kigoma Region, western Tanzania. Thematic analysis of emerging themes identified barriers affecting access to locally available malaria services and interventions.

**Results:**

Inclusion of persons with disabilities in planning, implementation and reporting of health issues in different malaria programmes was reported to be limited. Persons with disabilities were unable to access malaria services because of different barriers such as the distance of the service provision sites, communication and information issues and a lack of financial resources.

**Conclusion:**

Persons with disabilities are widely excluded from malaria care provision across the entire health services paradigm, impacting access and utilisation to this vulnerable population. Barriers to malaria service access among persons with disabilities were physical, attitudinal, financial and informational.

**Contribution:**

The findings of this study identify that malaria intervention stakeholders need to take a holistic approach and fully involve individuals with disabilities at all levels and scope of malaria service planning and provision.

## Introduction

Malaria infection remains a major public health challenge to most sub-Saharan African countries (World Health Organization [WHO] 2021). About 247 million cases and 619 000 deaths because of malaria were registered globally in 2021, the majority being from sub-Saharan Africa (WHO 2022b). Tanzania is 1 of 11 countries with the highest burden of malaria (WHO 2022b). Recent reports on malaria status in Tanzania showed that malaria is still the leading cause of morbidity and mortality, with notifications of infection transmission resurgence in some areas (Chacky et al. 2018; Finda et al. 2018; Insehngoma et al. 2018; Mapua et al. 2022; Mitchell et al. 2022; WHO 2021). Kigoma Region is one area in Tanzania contributing to the observed high burden of malaria, as it has a very high transmission risk (Runge et al. 2022).

In Tanzania, there are 3.6 million persons with disabilities, representing 8% of the population (Tanzania National Bureau of Statistics [TNBS] 2002; WHO 2022a). Among persons with disabilities, the majority have a physical impairment (47.9%), whereas 16.3% have intellectual impairment, and approximately 13% have multiple forms of impairment types (TNBS 2002). According to recent statistics (2023) from the Comprehensive Community-Based Rehabilitation in Tanzania (CCBRT), persons with disabilities have been reported to often live in extreme poverty because of the challenge of securing a steady income amid widespread exclusion from the workplace, either through discrimination or inaccessible work environments. Only 3.1% of individuals with disability in Tanzania receive income from paid employment resulting in this group experiencing extreme levels of poverty (CCBRT 2023). In this context, persons with disabilities are subjected to increased vulnerabilities.

The WHO calls for an urgent need to scale up disability inclusion in all levels of the health system and in the provision of health services (WHO 2021). In sub-Saharan Africa, persons with disabilities usually face a multitude of challenges that may hinder them from fully accessing and receiving healthcare services; such challenges include discrimination, a lack of knowledge on health issues, ineffective communication between persons with disabilities and healthcare providers, a lack of financial power, security and privacy problems (Baratedi et al. 2022). In most cases, persons with disabilities are often of low social economic status and the most marginalised in society (Tesemma & Coetzee 2022), impeding efforts to access malaria prevention and treatment services and therefore increasing the risk of morbidity and mortality because of delayed and untimely malaria treatment (Chuma, Okungu & Molyneux 2010; Ingstad et al. 2012). One of the main barriers against malaria prevention and control in Africa is inadequate information regarding means of malaria transmission, prevention and treatment among persons with disabilities (Ingstad et al. 2012). Factors that hinder persons with disabilities’ access to malaria services, as well as their related prevailing challenges and existing opportunities remain largely unexplored in both Tanzania (Rohwerder 2020) and other malaria endemic countries. In view of ensuring equitable health services to all citizens in Tanzania amid resource constraints, understanding the barriers affecting access to malaria services among persons with disabilities is pivotal.

Tanzania is one of the countries in Africa that has made a commitment to support the healthcare needs of persons living with disabilities. The *Tanzania Disabilities Act* (2010) clearly states that persons with disabilities have undisputed rights to receiving and benefiting from available healthcare services, such as treatment, therapy and rehabilitation. However, implementation of such a commitment has not been well-documented.

This study examined the present status of malaria service access and linked barriers among persons with disabilities in western Tanzania. The study aimed at examining the experience of persons with disabilities in accessing malaria services, identifying both enabling factors and barriers for persons with disabilities to access both facility-based and community-based malaria services, and knowing the extent to which persons with disabilities are involved in planning and monitoring malaria activities.

## Research methods and design

### Study design, study settings and study population

This was a qualitative case study focusing on social, cultural and health system factors associated with the inclusion of persons with disabilities in malaria services. A rapid qualitative case study was carried out to provide a quick understanding of the disability inclusion in malaria service provision in Kigoma Region, western Tanzania. Kigoma Region borders Burundi, Rwanda and the Democratic Republic of Congo (DRC). The region is one of the sentinel surveillance sites under the National Malaria Control Program, chosen because of the existence of very high malaria transmissions (Chacky et al. 2018). Two districts, Kibondo and Kakonko, with a high rate of disability (6.57% and 8.47%, respectively, versus 4.1% for the Kigoma region) (Tanzania Disability Monograph 2016) and relatively high malaria positivity rate (above 30%) were purposively selected.

According to the Tanzania 2020–2025 Malaria National Strategic Plan (MNSP), malaria statistics are obtained through public and private points of care where suspect patients are registered and tested (Tanzania Ministry of Health [MOH] 2020). Malaria data are then reported to the respective district and region via well-entrenched Health Information Management Systems (HMIS) – a system designed for health information collection, storage, analyses and evaluation of health-related data from health facility to district, regional and national administrative levels (Mboera et al. 2021). Malaria data in Tanzania are not reported according to impairment status. Malaria data available are disaggregated by sex and age groups.

Key informants included district malaria managers (coordinators), health officers, members of the associations for persons with disability in the respective districts, non-governmental organisation’s (NGO’s) representatives and malaria intervention programme officers.

### Data collection

Disability inclusion in malaria service provision is multidimensional. To gain a comprehensive understanding of the situation, three different qualitative approaches, namely, key informant interviews (KIIs, *n* = 12), in-depth interviews (IDIs, *n* = 18) and focus group discussions (FDGs, *n* = 10) were conducted to saturation of emerging themes. We also reviewed meeting minutes from organisations of persons with disabilities where available. Persons with different types of disabilities (gender and type of disability) were considered. Persons with disabilities in Kigoma Region are registered in civil organisations based on their specific impairments. Such classifications of disabilities were used to reach and recruit study participants.

In selecting study participants, firstly, civil organisations involving persons with disabilities were identified. Secondly, to ensure representation of all impairment, a list of individuals with disabilities was used to select participants with the help of community development and social welfare officers. Thirdly, respondent-driven sampling was also deployed to maximise heterogeneity of the study participants. Each of the KIIs, IDIs and FGDs took at least 30–45 min. Swahili language was used during the interviews because it is a common language to most persons with disability and majority of the Tanzania population. All interviews were tape-recorded, transcribed verbatim and later translated from Swahili to English and then back to Swahili to ensure a common understanding. Data obtained at every interview were used to guide coding rather than imposing a coding scheme. Each interview was moderated by two people experienced in qualitative research and a research assistant taking notes to enhance transcriptions accuracy of the recorded information. For KIIs, the district malaria coordinators, district social welfare officers and district health officers, NGO leaders and malaria programme officers were purposely selected and recruited to participate. Moreover, some health officers and healthcare workers at the ward level were also purposely selected to give their views on the subject in question.

### Document review

Meeting proceedings documented from selected village health committees, ward development committees and meetings from organisations of persons with disabilities at the district level were requested and reviewed where applicable, with the aim of reviewing any documentation related to malaria interventions and/or services among persons with disabilities. Moreover, the council comprehensive health plans (CCHPs) and malaria morbidity profile for both districts were also reviewed.

### Ethical considerations

The study protocol to conduct this study was reviewed and approved by the Joint Research and Ethics Review committee which comprises of two institutions, namely Catholic University of Health and Allied Sciences and Bugando Medical Centre Research and Ethics Review Committee (CREC/622/2022). Permission to conduct this study was also granted from respective authorities in Kigoma Region and Kibondo and Kakonko districts. Permission from respective persons with disabilities civil society organisations was also granted. All study respondents were informed of the purpose of the rapid qualitative survey, and written informed consent was provided via signature or thumb print. In instances where persons with disabilities were unable to read and write, the consent form was read to them, and they were allowed to ask questions. An impartial witness from the local community was present during the informed consent process to ensure that persons with disabilities were not coerced to participate and that their participation is completely voluntary. Persons with disabilities who were younger than 18 years of age provided assent, and their parents or guardians provided informed consent. The witness also signed the informed consent form and the assent form. Special codes instead of names were used as identification codes during interviews.

### Data analysis of key informant, in-depth interviews and focused group discussions

Audio files from KII, IDI and FDGs were transcribed verbatim and later translated into English. To verify and double-check the translated documents’ consistency, back translation was performed. Thematic content analysis was used to analyse the translated scripts (Marks & Yardley 2004). The following procedure was followed:

- Data organisation; to facilitate comprehension of concepts, information from KIIs, IDIs and FGDs transcripts were arranged under each topic and specific question separately to enable picking up concepts.
- Finding and organising concepts; identification of recurring concepts, and patterns was undertaken.
- Creating codes; coding was carried out and allocated to categories inductively; two people coded separately. Informants Interviews, In-depth interviews, and Focus Group Discussions are presented using special coding keys (See Appendix 1).
- To minimise inter coder variability, codes with a good degree of agreement were used.
- Building themes from available data: different concepts were collapsed into one theme. The arising themes were described with their relationships.

Of the six organisations of persons with disabilities contacted, two did not have meeting minutes, three organisations shared meeting minutes, but no issues regarding malaria services and/or interventions in any form were documented.

## Results

### Demographic characteristics of the study participants

Four FGDs involving persons with disabilities, 20 KIIs (which included the district medical officers, social welfare officers, malaria coordinators, leaders for persons with disabilities, healthcare workers, NGOs’ leaders and programme officers), and 41 IDIs were conducted in Kakonko and Kibondo. Most of the persons with disabilities (*n* = 21, 47.7%) had either never gone to school or did not complete primary school. The most prevalent disabilities among those who were recruited for the study included deafness, locomotors, albinism, autism, blindness and mental illness as detailed in Table 1. Categorisation of the key informants who participated in the current study are detailed in Table 2.

**TABLE 1:** Demographic characteristics of the persons with disabilities (*N* = 44) who participated in the rapid qualitative survey.

Variable	Category	*N*	%
**District**	Kakonko	22	50.0
	Kibondo	22	50.0
**Sex**	Male	26	59.1
	Female	18	40.9
**Marital status**	Married	26	59.1
	Single or separated	18	40.9
**Education**	Never attended school	11	25.0
	Did not complete primary school	10	22.7
	Completed primary school	19	43.2
	Secondary school	4	9.1
**Occupation**	Self-employed	26	59.1
	Unemployed	16	36.3
	Minor (below 18 years)	2	4.5
**Disability type**	Locomotor disability	27	61.4
	Blindness	6	13.7
	Deafness	6	13.6
	Autism	2	4.6
	Albinism	2	4.6
	Mental illness	2	4.6

*Source:*

**TABLE 2:** Key informants involved with persons with disabilities (*N* = 20) who participated in the rapid qualitative survey.

Categories of key informants	Sub-total	Geographical zone					
		Regional		Kibondo District		Kakonko District	
	*N*	*n*	%	*n*	%	*n*	%
Malaria coordinators	3	1	5.0	1	5.0	1	5.0
Health officers	6	2	10.0	2	10.0	2	10.0
Community health workers	5	-	-	3	15.0	2	10.0
Leaders of persons with disabilities	2	-	-	1	5.0	1	5.0
Social welfare officers	2	-	-	1	5.0	1	5.0
Community development officers	2	-	-	1	5.0	1	5.0

### Identified themes in a rapid disability inclusion barrier analysis survey on malaria services in Kigoma

Thematic content analysis of the interview data revealed four main themes and several sub-themes as presented in Box 1 and discussed thereafter based on the objectives of the study.

BOX 1 Emergent themes from thematic content analysis.

**Themes**

*Awareness, experiences, and enabling factors among persons with disabilities in accessing malaria services (IDIs [n = 12], KIIs [n = 6], FGDs [n = 5])*

- Some respondents were aware of malaria disease
- Information access barrier associated with illiteracy levels among persons with disabilities

*Barriers that hinder persons with disabilities to access Malaria services (IDIs [n = 13], K IIs [n = 8], FGDs [n = 7])*

- Non-inclusive malaria services for persons with disability
- Struggle with locating malaria services when unavailable at health facilities.
- Existing communication barrier for persons with hearing as well as those with mental impairments with healthcare providers.
- Exclusion of persons with disabilities in free preventive malaria services (non-inclusion in subsidised malaria services) at health facility level.
- No special window – a dedicated place for special groups such as persons with disabilities when accessing malaria services.
- Inadequate comprehension ability for malaria services uptake among some persons with disabilities.
- Expenses to cover malaria medication was a challenge.
- Distance to reach nearby health facilities.
- Transportation to health facilities and the health facilities themselves where malaria services are offered are not accessible to persons with disabilities because of financial constraints.

*Stigmatisation of persons with disability in accessing malaria services (IDIs [n = 10], KIIs [n = 3], FGDs [n = 4])*

- Some families hide persons with disabilities in their homes preventing them from getting malaria services that are offered at the household level.
- Healthcare providers are unaware of communication skills to use with persons with disabilities, such as sign language.
- Staff attitudinal behavioural to persons with disabilities.
- No priority given to individuals with disabilities once accessing healthcare services.

*Disability inclusion in existing malaria-related programmes (IDIs [n = 7], KIIs [n = 4], FGDs [n = 5])*

- Non-inclusion of persons with disabilities in malaria-related system and policies.
- Health insurance – an insurance product which reimburses the expenses incurred by the care provider of the insured individual directly – coverage and scope is low among persons with disability.

KII, Key informants interviews; IDI, in-depth interviews; FDG, focus group discussion.

### Awareness, experiences and enabling factors among persons with disabilities in accessing malaria services

The respondents in Kibondo and Kakonko districts had different experiences in accessing malaria prevention, promotion and curative services. Most of the respondents said there was a special consideration for the elderly, children and pregnant women, while persons with disabilities were left aside. One of the respondents with disability from the IDI mentioned:

‘I’ve never seen a person with disabilities being given a special priority contrary to the old people. In most cases old people have been given special priority in receiving treatment.’ (IDI37, 18YRS, male)

This information is supported by one of the key informants who added ‘… those services are provided in general, there is no specific or special consideration for persons with disabilities or outreach services to reach those with disabilities’ (KII30, 37YRS, male):

‘We have heard the Malaria treatment plan for all in the media, but it is not implemented because it aims to treat children and pregnant women and not those with disabilities.’ (FGD20, 30YRS, female)

It also emerged that it was difficult for persons with disabilities to access malaria services because they were not aware of what kind of malaria services were available in their communities or nearby healthcare facilities. This was supported by one of the respondents:

‘The information we get from the radio and newspapers is that Malaria is caused by the presence of water in the pools … but when we become sick, we do not know where to run to because at the hospital we don’t get proper care thus we go to the pharmacy to borrow some medication.’ (FGD20, 48YRS, male)

Regarding the distribution of nets, most of the respondents said that it was difficult to get insecticide-treated nets (ITNs) if you do not have children at school or pregnant wife:

‘In terms of spraying, they do not discriminate, if you can take your belonging and all individuals around outside the house, but in terms of mosquito nets, clinics provide services to pregnant mothers and school children only.’ (IDI41, 42YRS, male)

Another one added that:

‘We rely on primary school children to be given the bed nets that we use, but as persons with disabilities, I have never received one.’ (IDI56, 28YRS, female)

However, even though bed nets are distributed, some persons with disabilities have different beliefs and perception about them:

‘… some people because they think that maybe bed net reduces male potency, sometimes people have different interpretations, especially when people give interpretations to those who do not have knowledge of medicine and prevention against Malaria. They do not investigate, because people have already used mosquito nets so we can use them, but we will not be able to have a case of this Malaria even though Malaria exists.’ (IDI2, 19YRS, female)

Others said the nets brought bed bugs and other discomforts:

‘They bring bedbugs, there is a certain period [*laughter*] President Kikwete gave aid in nets, as a result a lot of people were attacked by bedbugs, many burned them, and they were afraid to use those nets.’ (FGD23, 28YRS, female)

Others added:

‘If it is still new, you sneeze frequently, I think they are affecting because you are sneezing frequently until you wash it, and the medicine is reduced, you must wash it before you use it.’ (ID112, 45YRS, female)

‘I sweat a lot sometimes when I use bed net.’ (IDI15, 63YRS, female)

However, others had positive opinions on mosquito nets and encouraged that people should be educated, as one said:

‘…. so a student who grew up in Kigoma who didn’t have lice or maggots, was not a student, but after sometimes we realized what cleanliness means until today … I think it is not true that bed nets bring bed bugs people should be educated about cleanliness.’ (FGD23, 30YRS, male)

Respondents understood that in all health institutions, including dispensaries, all malaria services are delivered free of charge, which includes testing, diagnosis and treatment. All medication of malaria should be given free to all citizens regardless of age groups; however, because of scarcity of medication, individuals with malaria must buy malaria drugs in the nearby pharmacy. As one said:

‘… the doctor will prescribe it for me, and I will take it to the store. At the medicine window, I will find that this medicine is not there, I go and buy it. That’s why I think it’s better to go to the street and buy at the pharmacy directly.’ (IDI16, 63YRS, female)

Another one added:

‘It is better to go to the pharmacy because when you arrive at the hospital, and there is no medicine, you find that you have wasted the time and costs of coming to the hospital, so it’s better to buy and take it without testing.’ (IDI12, 45YRS, female)

It was also established that persons with disabilities get proper care most of the time. They do get exemption for treatment and sometimes they are given priority in service delivery; however, there is no special window – a dedicated place for special groups such as persons with disabilities when accessing malaria services – in a particular health facility; therefore, they are sometimes asked to queue at the window of the older people, to ease delivery of services for them:

‘Persons with disabilities have the opportunity to be treated, when they show up, we usually treat them with exception … they get an opportunity because all their treatment from tests to medicine, is free but they are also given priority to be treated first if they are in a queue, but having a window for persons with disabilities, is difficult so we connect them to the window for the elderly due to shortage of rooms.’ (KII4, 38YRS, male)

Some of the health providers were aware that persons with disabilities are a vulnerable group who need special consideration as one mentioned:

‘Persons with disabilities are my customers because they are a special group. An individual with disabilities is a human being like any other human beings because he gets the same rights as anyone else and what we are looking at is that one gets the right and safe services because he should not be isolated due to his or her disability.’ (KII40, 33YRS, male)

Another one added that:

‘The majority of those who do not have health insurance are given waivers [*social welfare*] regardless of age, whether young or old.’ (KII21, 38YRS, female)

Some persons with disabilities acknowledged to be given special consideration as one person with visual impairment mentioned:

‘Opportunities, sometimes [*probably*] you are treated free, or may be when you arrive at the health centres you will be the first to be treated, you don’t que, when we go to the hospital, we have been given priorities of being the first ones to receive treatment.’ (IDI38, 26YRS, male)

Another enabling factor for persons with disabilities is the presence of relatives or family members who help them to access malaria services. One respondent said that ‘my husband is involved when it comes to paying for my treatment’ (IDI53, 38YRS, female):

‘When my children find work, such as fetching water or doing domestic work, they help me buy medicine.’ (IDI13, 45YRS, female)

Persons with disabilities can directly receive information about malaria services through radio, neighbours or campaigns as one said in a group discussion:

‘On the availability of malaria information for us, people who live near the town can hear the council cars pass while announcing, but for persons with disabilities who live in the villages, they don’t get any information because even the cars can’t get there, they don’t have a radio and they don’t read newspapers. The government or health workers should reach out to those in the villages and provide health education.’ (FGD20, 30YRS, female)

‘There are neighbours who always come and tell us that there are mosquito nets today, they are bringing medicine and they are coming to spray, so get prepared.’ (IDI18, 82YRS, female)

Also, the presence of sign language teachers helps individuals with hearing disability:

‘There is a school that teaches them [*the school for the deaf that exists in Kanyamahela*] many things including health education … most people send them there and the care givers are also taught practical sign languages.’ (KII21, 38YRS, female)

Sometimes persons with disabilities are given opportunities to perform leadership roles and community engagement activities as one malaria coordinator from Kibondo said:

‘They become very less suspicious and build confidence in the sense that the group involves Community health workers [CHWs] – lay members of the community who work either as volunteers in association with the local health care system against malaria … we are still looking for the best way to increase their participation through other means, meaning that even those who come in groups as CHW who have disabilities, we are getting their opinion as well.’ (KII31, 41YRS, male)

### Barriers that hinder persons with disabilities to access malaria services

#### Sub-theme: Physical barriers

Physical barriers are a major factor that hinder accessibility of malaria services, as some health facilities are built far away from people’s residences; one of the respondents said:

‘The obstacles I see is because there are some persons with disabilities who cannot walk, now you find that they come to hear that there is a free service provided to persons with disabilities, so the biggest problem is getting fast transport.’ (IDI41, 42YRS, male)

It was also added that:

‘I can spend about 40 minutes on foot in terms of transportation like 10 minutes by motorcycle … in the rural areas the fare is 2000 but in the district the fare is 5000 to 10000 shillings.’ (FGD20, 28YRS, female)

Also, physical barriers can have an impact on indoor residual spraying as Indoor Residual Spraying (IRS) safety officer said:

‘IRS is a poisonous exercise, so we can’t spray in the presence of people or things, they must take all things out. Sometimes we encounter a challenge, a person with a disability or a patient is unable to give or take out their belongings, we fail to execute the exercise, we must continue to other premises.’ (KII52, 35YRS, male)

It was also found out that persons with disabilities may not get emergency treatment in case of a sudden illness because of infrastructure as a nurse from Kibondo said:

‘When individuals with disability are at home, help is not enough, for example, an individual with disability who use tri-cycles until they find people to push them, so if he does not get help, it is a challenge, it means that even if he is caught by a sudden illness, he cannot rush to hospital quickly due to his disability.’ (KII21, 38YRS, female)

#### Sub-theme: Communication barriers

In the KIIs, sign language emerged as one of the communication barriers among persons with hearing impairments versus healthcare providers during malaria services provision. For instance, healthcare providers face language barriers when it comes to communicating, ‘those who have some disabilities such as those unable to speak … are challenging to treat because we cannot understand each other’s language’ (KII3, 30YRS, male).

It also emerged that sign language is taught but many people do not go to special school to learn it. This poses a challenge in accessing healthcare services even in the presence of sign language professionals as evidenced by one of the professionals:

‘The challenge is communication for the deaf in the signs. There are signs that are standard, Tanzania sign language, and there are signs that are normal local. Now there is a big barrier between Tanzania sign language and normal sign language because they vary from person to person, for example you find a certain sign that he means, you don’t understand it and the larger society still has no understanding of sign language, so you find that when these children with special needs want to express themselves, it becomes a challenge to be understood in the offices or institutions they go to and sometimes you may be called a professional should also do sign language interpretation, but you also find that the person has signs that you are not familiar with. So, sign language develops as a language. It needs time to stay with the community, especially for people who did not come to our school where Tanzania sign language is taught.’ (FGD6, 45YRS, male)

Other participants admitted that they are not able to get any health-related information because of their disabilities, as one said, ‘I don’t get any information because first of all I can’t hear, so maybe there would be leaflets for those of us who can’t hear’ (FGD23, 37YRS, male).

#### Sub-theme: Financial barrier

Persons with disabilities are more likely to experience poverty, which poses financial barriers to accessing health services; one of the malaria coordinators mentioned:

‘They do not have the ability to do economic work like other people, and if we look at the second group, those with mental disorders, those with this type of disability are at greater risk because most of them will be born normally, there is none, they need more attention but we still don’t have a specific program for them.’ (KII31, 41YRS, male)

Most of the respondents admitted that they cannot afford health services because they have a disability and so they are more reliant on help and support from others in most aspects of life. As explained by one respondent who said:

‘He can’t afford it because he doesn’t do any work, he stays at home.’ (IDI17, 68YRS, female)

Another participant with disability added that:

‘We individuals with disability do not have the income, so when we get sick at night, getting transportation becomes a problem … a motorcycle that will bring you to the health centre, costs 15000.00 and a car cost 20000.00 now and for us as individuals with disability cannot afford. I was asking you to at least help us find a way to get transport when we are being taken to the hospital or clinic.’ (FGD20, 48YRS, male)

Others stressed that services are expensive and went further to ask the government to provide special insurance for them:

‘It is difficult for us persons with disabilities to pay for the services, because most of us do not have special jobs, so we would like the government to see how it can help us.’ (FGD7, 40YRS, male)

‘When you go to the hospital without health insurance for the treatment of malaria and other diseases, if you go for tests, it is expensive because typhoid costs 6000.00 shillings, now if you don’t have enough money, you will only have to take one test for example malaria, then that becomes a problem, we ask you to help us.’ (FGD20, 39YRS, male)

‘We don’t have insurance specific for persons with disabilities, so when we get there, it becomes difficult to get services, so we were asking the government to set aside a window for persons with disabilities like the elderly and given health cards.’ (FGD20, 48YRS, male)

### Stigmatisation to persons with disabilities in accessing malaria services

Some respondents reported facing some form of stigmatisation when seeking healthcare in health facilities and in the society. Some who had faced stigma added that not all people show stigma to the persons with disabilities, as he said:

‘Except to be looked at badly, this has happened to me, for example, one person told me that he was serving others, he left me there and his colleague came and told him that why have you not taken care of him and you have served others, you are doing wrong, so he took me and went to serve me.’ (IDI56, 28YRS, female)

Leaders of organisations pointed out that some persons with disabilities are left behind because some members of the society view them as a burden and hide them:

‘Some people see individuals with disability as a burden, so they don’t appreciate them very much. We have been witnessing and hearing on various media that some individuals with disability have been found hidden, this is a challenge to them, and it can easily lead to lack of services. That’s why I advised the community to see persons with disabilities as people like them, people like everyone else and they should be able to get the services they deserve without discrimination and without stigmatizing them … I think the biggest thing is to be seen as a burden in that society … the basic thing is that people can be educated to remove the concept of discriminating persons with disabilities.’ (KII4, 38YRS, male)

Persons with disabilities experience delays in being taken to the hospital unless they are severely unwell as one clinician said:

‘The challenges persons with disabilities face is that a person may have all the symptoms of malaria, but he or she is not brought to the hospital on time … until the patient is completely overwhelmed and this is because most caregivers spend most of their time in the fields.’ (KII3, 30YRS, male)

Another malaria coordinator said:

‘The public’s understanding is limited so the support they get when they get sick is small, that’s why until they get really sick, they are brought to health care centres.’ (KII30, 37YRS, male)

Health provider’s attitude and behaviour can also be a major barrier for persons with disability to access malaria services. Some respondents reported that they faced some bad attitudes from health providers when they went for malaria treatment as no special services was offered to them despite their disabilities:

‘When you get to the hospital, if you don’t have insurance, they tell you to bring 3 thousand shillings and if you don’t have the 3 thousand shillings you can leave without even getting medicine.’ (FGD7, 49YRS, female)

‘At other times you may arrive, and they start insulting you and if you get there I give up, other times you find that he is angry, he pretends to be busy and doing his work and when he decides, he comes to serve them and he does that for all persons with disabilities and those without disabilities.’ (IDI8, 35YRS, female)

It was also evident that there was a lack of inclusion and/or involvement of persons with disabilities in planning and monitoring, implementation and provision of malaria services in the surveyed districts. Also not knowing the exact number of persons with disabilities makes planning and allocation of resources difficult. It was also suggested that the government health sector budget should be increased to avoid the tendency of persons with disabilities to buy medicine, as the district medical officer said:

‘It is difficult to know the number of persons with disabilities, this makes planning difficult because their number is not very clear, so your plan can be less or beyond the target level. But also knowing the different types of disabilities that people have becomes challenging, so it can cause a problem in planning what should be done to ensure that the service includes people with various disabilities during the installation, implementation and monitoring, I think there should be a good plan on how to identify persons with disabilities in different areas, maybe starting from the neighbourhood level upward, with cooperation from different offices like social welfare, these will have their number and the type of disability they have so that it can be easy to make plans to help them.’ (KII4, 38YRS, male)

Another respondent insisted, ‘I would ask that we should be involved in malaria services provision’ (IDI41, 42YRS, male).

The review of the CCHP – a guideline that entails a consolidation of Council Health Management Team and health facility plans so as to maintain and improve the health provision of promotive, preventive, curative and rehabilitative health and social welfare services and make it accessible, affordable, effective, equitable and of good quality (CCHP 2011) – found the existence of various community-based malaria interventions and services including malaria test and treat, mosquito net mass distribution and community awareness campaigns. Another activity was the implementation of the indoor residual spray programme in Kibondo District. Unfortunately, none of the two councils had a special programme to address malaria services and interventions that was dedicated to persons with disabilities. Like many other programmes, the focus was mainly on pregnant mothers and children under the age of 5 years.

### Disability inclusion in existing malaria-related programmes

Existence of relief services, leaders, stakeholders and programmes in Kibondo and Kakonko Districts such as Tanzania Social Action Fund (TASAF), Help Age International and Catholic Relief Services (CRS) has been involved with persons with disabilities and even helped a lot of persons with disabilities in mitigating some of the health challenges as one respondent said:

‘At the moment we have two organisations that are helping us, the first is ABT Associate through USAID who are doing the IRS exercise for us, the second is MSF through the Nduta camp with whom we also collaborate on bio-larviciding although they are concentrated in the areas surrounding the refugee camp.’ (KII31, 41YRS, male)

‘I also link institutions including health care centres, but there is also a national Malaria program, which is also a government institution, and there is a PMI vector link, they were linking us to the CRS.’ (KII30, 37YRS, male)

However, there are few special programmes for malaria that are designated to help persons with disabilities as most of the respondents said and observed:

‘The government should strive to improve the infrastructure throughout the country, district hospitals should have a window for persons with special needs and there should be expert doctors who have studied units for the blind, and deaf persons. Those with special needs need to be conveyed the message so that they can help them so that their health can improve, the government should also create infrastructure to enable these persons with special needs who are unable to walk from where they reached to find a place to live, but I believe that the government allocates the budget properly.’ (FGD23, 41YRS, male)

One of the participants added, ‘I think the Government should help us on the provision of appropriate health services, especially people like us with children with disabilities, we have countless challenges’. (IDI27, 38YRS, female).

It was observed that there were various malaria-related policies at the district level; however, none were centred on persons with disabilities; one informant explained:

‘The government should put efforts on knowing that there are special groups of persons with disabilities who need access to services in the areas where they live. If the government puts pressure on it, I think those barriers may disappear and we may find ourselves not being discriminated against.’ (FGD7, 42YRS, female)

‘We rely on the children of the primary school to be given the bed nets that we use, but as persons with disabilities, we have never received mosquito nets.’ (IDI56, 26YRS, female)

‘I would like the government to help us with the provision of nets and spraying, that is, for example, maybe there should be nets distributed special for us persons with disabilities.’ (IDI56, 26YRS, female)

A majority of persons with disabilities suggested that the system should consider them in terms of treatment and services provision, like all other groups through providing them with health insurance:

‘We don’t have health insurance for persons with disabilities, so when we get there, it becomes difficult to get services, so we were asking the government to set aside a window for persons with disabilities like the elderly and health cards and we should be given.’ (FGD20, 48YRS, male)

It was concluded that the district councils were ready to work and cooperate with the government and stakeholders in the fight against malaria as one said:

‘Kibondo is the district council itself is in the front line supporting the Ministry of Health which brings us the tools, but we have had the MSF organization, they are participating in the bio-larviciding campaign, for the neighbouring areas, we have been collaborating with Tanzania Vector Control Agency which is an organization that deals with statistics and research against Malaria but we were also collaborating with NIMRI to research the potential of mosquito nets and drugs and the type of mosquitoes we have and lastly we were collaborating with USAID people to distribute mosquito nets through lion net.’ (KII31. 41YRS, male)

Additionally, education is one of the adopted means to fight malaria in the district as one healthcare provider explained:

‘… Along with that we provide education for them regarding problems that a person may experience if they suffer from Malaria regularly, such as anaemia and splenomegaly. We think this will create fear among them and they will take initiatives to protect themselves from Malaria. Even at home they should check if their houses are surrounded by bushes, they should try to clean the house as well and sleep in a mosquito net, and if the mother is pregnant, she should make sure that she follows the procedure of taking SP medicines so that she cannot give birth to a child with Malaria.’ (KII21, 38YRS, female)

Persons with disabilities are a special group and see themselves as more vulnerable and need special attention. It emerged that the use of community health workers (CHWs) was vital as they are familiar with and have built trust with such an individual and sometimes there are CHWs who are among the persons with disabilities. One of the informants mentioned, ‘they become less suspicious and build confidence in the sense that the group involves CHW who are working with the community organization against malaria …’ (KII31, 41YRS, male).

During data collection, enumerators learned that organisations of persons with disabilities have a networking system that enhances access to those in remote areas.

## Discussion

The Alma-Ata Declaration (WHO 1978) proclaimed primary healthcare as the means for achieving ‘Health For All’, also the universal health coverage (WHO 2022b) with the stance that all people have access to the full range of quality health services they need, when and where they need them, without financial hardships. This Declaration has influenced the reorganisation of the health systems in all countries including Tanzania. National policies are based on effective, cost-efficient primary healthcare strategies that entail universal health coverage, patient-centred approaches and demand-driven health policies. Persons with disabilities, like any other individuals, have the right to good health and equality in access to malaria health services (WHO Policy on disability 2021a). Therefore, it is important to understand their existing barriers to health services to identify approaches to improve service access. This study revealed several enabling factors and barriers for persons with disabilities to access both facility-based and community-based malaria services.

According to the Tanzanian Disability Survey (2008), it is estimated that more than 20% of persons with disabilities encounter some barriers when accessing health services including malaria services. Furthermore, there is no full involvement of persons with disabilities in planning, implementation and reporting health issues in different programmes (WHO 2022b). As respondents observed, multiple barriers exist for persons with disability to access malaria services. These range from a lack of information to the actual cost of services, increasing health disparities among persons with disabilities: physical barriers that prevent access for persons with disabilities to health clinics and hospitals; informational barriers that prevent access for persons with disabilities to health literacy and information brochures and leaflets on health promotion, prevention and protection; attitudinal barriers, which give rise to discrimination that can have severe implications for the rights of persons with disabilities.

Persons with disabilities share different experiences while in need or when seeking malaria services. These experiences are unique and peculiar to each individual person. The degree of experience may differ depending on the different types and needs of everyone with disability. Our findings that such individuals are not given service accommodations could be a demotivation in seeking healthcare among persons with disabilities. For instance, there were no dedicated places for persons with disabilities while accessing healthcare services, unlike older people. Persons with disabilities struggle to reach such services taking them longer as compared with a normal person, yet they have to look for their day-to-day basic needs; this is supported by data from sub-Sahara, which indicate that almost all national malaria control programmes in Africa give little or no special attention to this population (Rohwerder 2020). Furthermore, studies in East Africa have shown that persons with disability are less likely to be covered by general public health interventions (Schenk et al. 2020) with reports from Tanzania revealing high exclusion of persons with disabilities from accessing healthcare services (Chuma et al. 2010).

The hardship may range from a lack of ramps, presence of steep ramps to rough pavements that hinder their movements to and around health facilities surroundings freely and independently. Other risks include inaccessible and unclean hospital toilets and non-adjustable hospital beds, to mention a few (WHO 2022b). We recommend efforts to integrate persons with disabilities within the special window for older people. On the one hand, without services tailored to their specific accessibility needs, persons with disabilities can be predisposed to higher chances of developing complications related to malaria infection as well as heightening the risk of mortality (Chuma et al. 2010; Ingstad et al. 2012; Torres et al. 2019). On the other hand, the western part of Tanzania has high mobility influx of people in search for gold using rudimentary methods with poor housing – artisanal and small-scale gold mining (ASGM). Areas with ASGM activities are reported to lack malaria prevention as well as treatment services making such economic activities a major driver in the increase in malaria to the general population (Castro & Peterka 2023). Landier et al. (2016) established the role and importance of malaria diagnosis in the efforts of treatment and elimination, where individuals who had limited or no access to diagnosis had poor prognosis and sometimes succumbed to death from the disease.

The lagging of malaria services among persons with disabilities is multidimensional, hence community engagement is vital. Community engagement is grounded in the principles of fairness, justice, empowerment, participation and self-determination while enhancing adequate community protection especially among the marginalised such as persons with disabilities (Eversole 2010). Implementation of community-based malaria programme targeting persons with disabilities, e.g. malaria test and treat programmes, yearly long-lasting insecticidal nets (LLINs) distribution, household IRS and awareness raising campaigns, is therefore recommended in these communities.

The findings of this study also indicate a lack of sign language and special communication assistance to persons with hearing impairment as well as those with sight impairment (Baratedi et al. 2022) across all the malaria scope of services limiting the type and kind of services they get. It emerged that medical staff, for instance, are rarely trained and/or have such training or expertise regarding sign language for people with hearing challenges.

In addition, a lack of information on malaria services is one of the reported barriers to access to services for persons with disabilities. There were neither government nor community efforts that were evident in the surveyed districts in ensuring persons with disabilities receive accessible health information. This study also indicates that affording to have and operate a means of information receiving devices such as radios and TVs to access information was a challenge to some of the persons with disabilities. Communication barriers were also reported when persons with disabilities visit health facilities trying to access malaria services.

There are reports that persons with disabilities who managed to reach health facilities could also be inclined to seek medication at a nearby private drug store or pharmacy as medication is frequently unavailable in most of the rural health facilities (Palmer et al. 2015; Torres et al. 2019), and persons with disabilities can hardly afford such expenses or purchases (Baratedi et al. 2022). It must be understood that a large number of persons with disabilities lacking financial resources coupled with a lack of privilege to such services puts malaria control efforts at risk (Palmer et al. 2015; Torres et al. 2019). For instance, because most persons with disabilities have limited financial resources, they therefore resort towards the practice of self-medication and over-the-counter drug acquisition (Palmer et al. 2015; Torres et al. 2019). This could be contributing to drug resistance and/or escalating the public health challenges against drug resistance. Our speculations are supported by findings reported elsewhere in Tanzania where self-medication and over-the-counter malaria medication have been reported as one of the reasons for potential drug resistance that could be currently affecting the entire community or would potentially affect such communities in the near future (Baratedi et al. 2022; Palmer et al. 2015; Torres et al. 2019).

With limited services and lack of privileges for persons with disabilities in accessing malaria services, possessing health insurance seemed to be important as an enabling factor. Study participants reckon that having health insurance allows them to access health services. Already Tanzania is finalising a legislation for health insurance for all. However, the cost for an individual is expected to be TZS 84 000.00 (equivalent to $35.00 USD) or TZS 340 000.00 (equivalent to $142.00 USD) for a family of six people. This remains to be a major barrier as most persons with disabilities live in extreme poverty and have failed to secure a Community Health Fund Card (Torres et al. 2019), which is reported to be worthy of TZS 10 000.00 per year in rural areas.

The *Tanzania Persons with Disabilities Act* (2010) commits the government to ensure access to health services including malaria services for all. In 2009, Tanzania government ratified the 2006 United Nations Convention on the Rights of Persons with Disabilities (UNCRPD). Article 25 of the UNCRPD urges States Parties to:

[*R*]ecognize that persons with disabilities have the right to the enjoyment of the highest attainable standard of health without discrimination based on disability. States Parties shall take all appropriate measures to ensure access for persons with disabilities to health services that are gender-sensitive, including health-related rehabilitation. (UNCRPD 2006:18)

Furthermore, the *Tanzania Disability Act* (2010) provides similar provisions. Even though the implementation of this commitment has not been fully evaluated, it provides an opportunity and an initial platform for stakeholders to push the government agenda to enhance access to malaria services among persons with disabilities, especially in areas with high malaria resurgence such as Kigoma in western Tanzania.

From a policy and legal point of view, the Government of Tanzania through the Ministry of Health has shown great commitment towards ensuring access to healthcare to persons with disabilities. The government has recognised and included the specific needs of persons with disabilities in policies and legal frameworks. The Health Sector Strategic Plan V states that the Government will ensure availability of essential primary healthcare services with acceptable quality standards throughout the country with respect to geographical, population, gender, disability and burden of disease. However, the great challenge remains on making its health programmes more inclusive to persons with disabilities by mainstreaming their needs.

Missing healthcare because of inability to afford it is another reported challenge. Most participants acknowledged not having sought care because of a lack of financial resources as a result of the affordability of the services and transport expenses to reach such facilities. Distance between home and the facility is among the challenges reported not only by persons with disabilities but also other people. However, because of their conditions, persons with disabilities are highly restricted from accessing health services as compared with other people. This coupled with financial inability to afford transportation costs has also been reported in Kenya, which neighbours Tanzania, as obstacles limiting accessibility of malaria health services (Otambo et al. 2023). In a recent report by Otambo et al. (2023), approximately 76.4% of the malaria febrile residents had delayed treatment because of their inability to afford malaria healthcare services. This, among other things, calls for immediate action aimed at resolving this financial barrier to encourage and enable persons with disabilities to access and afford malaria healthcare services.

Social and communal networks and non-governmental organisations emerged as important groups in supporting persons with disabilities to access health services, as coping strategies, and support for persons with disabilities. However, government support for persons with disabilities was found to be limited. Most of the local support from the government is limited to the elderly, pregnant women and young children. Some participants reported to have received assistance from their nearby relatives. Insecticide-treated mosquito nets, for instance, are distributed in schools (i.e. *you get one if you have a school going child*), given to pregnant women (*you get one if you are married*) and given to younger children below the age of 5 years (*you get one if you have an under-five child*); in this regard, persons with disability are not included in the ITNs distribution programme unless one is pregnant and/or has a child going to school. There is a need to push the agenda in malaria intervention programmes to consider including persons with disabilities as one of the special groups benefiting from free ITNs distributions. One of the limitations to this study is failure to assess the uptake and use of the existing malaria services such as ITNs among persons with disabilities. A follow-up study should examine the uptake and use of malaria services among persons with disabilities.

Healthcare worker’s attitude and behaviour towards persons with disabilities was different and inimitable to each participant. In some areas, negative attitude of healthcare workers towards persons with disability emerged in some of the interviews and discussions. We could not validate such contentions, as an observation study was needed. It is nevertheless important to sensitise healthcare workers regarding the rights of persons with disabilities and how they should be treated in accessing not only malaria services but also other health-related services. Generally, the findings of this rapid survey are supported by a previous Tanzania disability survey (2008), conducted in the Southern Part of Tanzania (in Iringa Region) that revealed five major challenges among persons with disabilities regarding access to health services in general, which included medical costs, inadequate medicine, communication barriers and inaccessible infrastructure.

Persons with disabilities in the western part of Tanzania report disparities in accessing malaria services. The 2022 *WHO Global report on health equity for persons with disabilities* acknowledges such disparities in health services among persons with disabilities that could be potentiating poor health outcomes among such individuals. In this study, we did not find any evidence for persons with disabilities being involved in planning and monitoring for malaria health services in the districts surveyed. The WHO calls for agent initiatives to address such health inequities among persons with disabilities to ensure the Global Sustainable Development Goals and global health priorities are attained (WHO 2022b).

Based on the current findings and existing evidence from Tanzania, the following recommendations should be considered in minimising existing barriers and leveraging opportunities. Firstly, from Tanzania’s policy and legal point of view, provide an opportunity for inclusion of persons with disabilities in accessing malaria services in the country. We therefore recommend policy advocacy on inclusion of persons with disabilities as a priority population by the country malaria control programmes, and reinforcing inclusive strategies and actions to benefit persons with disabilities in need of malaria services. Secondly, the attitudinal barriers at the health facilities should also be addressed. There is a need for provision of training of health workers on disability etiquette, the rights of persons with disabilities, communication tips and other accommodations to improve access to malaria services among persons with disabilities. Thirdly, the special window for provision of services for older persons can expand the scope to include services for persons with disabilities; this can also be integrated in the District Health Information System (DHS2) to provide the number and health issues involving persons with disabilities that is currently lacking (Braa & Sahay 2017). Fourthly, regarding experiences on malaria services among persons with disabilities, there is a need for malaria awareness campaigns on transmission, symptoms and signs and health-seeking behaviours to be implemented by using community health workers and peers’ educators who themselves have disabilities as part of the intervention initiatives. Fifthly, policies supporting special groups such as school-aged children, under-five children and pregnant women are provided with free ITNs. It is on this basis that persons with disabilities can be included among the beneficiaries for ITNs through their existing networks and social groups. Sixthly, sensitisation of Districts Community Development and Social Welfare Departments on provision of payment waiver for the persons with disabilities regarding treatment payment for persons with disabilities. Seventhly, as there was no agenda for reporting malaria issues in the minutes reviewed, there is a need to initiate capacity-building programmes for civil society organisations for persons with disabilities to enable them to include malaria issues in their meetings. Lastly, there is a need to strengthen the networks of persons with disabilities at the district level to improve their specific health problem-solving skills and to be able to reach those at the very low levels and harmonise the specific services provided in urban areas.

## Conclusion

Widespread exclusion of persons with disabilities in malaria services provision exists across the entire health services paradigm affecting access and utilisation to such a vulnerable group. Information about malaria services to persons with disabilities was evidenced to be minimal and/or lacking. Involvement of persons with disabilities in planning, implementing and reporting health issues in relation to malaria in different programmes has been lagging. Generally, barriers to malaria service access included physical, attitudinal, financial and informational. To ensure equitable malaria services and interventions, the barriers should be minimised by deliberate efforts tailored to persons with disabilities during planning and implementation of health services at the health facility and community level.

Opportunity for improving the situation in policy and legal aspects, use of CHWs and civil society organisations for persons with disabilities exist. The existing opportunities for inclusion require coordinated innovative approaches such as the use of community ambassadors for persons with disabilities as well as strengthening community networks among individuals with disabilities.

## Figures and Tables

**FIGURE 1-A1:**
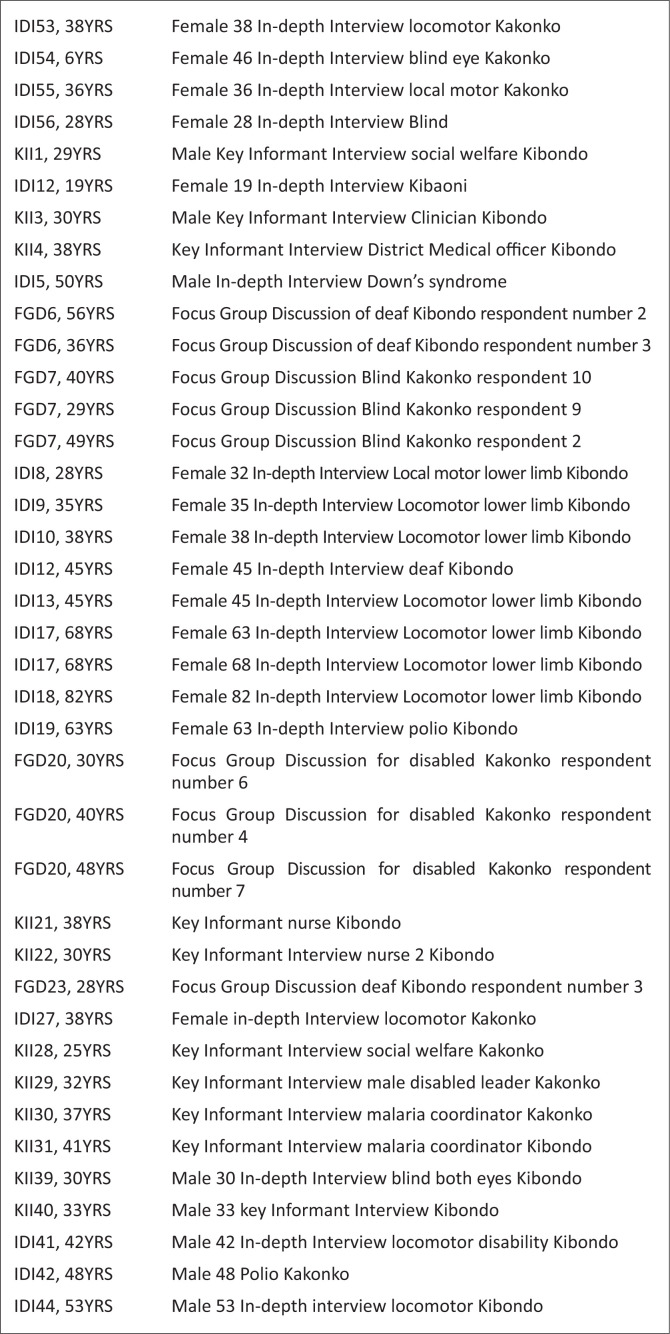
List of the coding keys used in the key informants interviews, in-depth interviews, focus group discussions.
